# Menin Maintains Cholesterol Content in Colorectal Cancer via Repression of LXR-Mediated Transcription

**DOI:** 10.3390/cancers15164126

**Published:** 2023-08-16

**Authors:** Thomas E. Nyul, Keely Beyries, Taylor Hojnacki, Rebecca Glynn, Kayla E. Paulosky, Anitej Gedela, Ariana Majer, Lily Altman, Kole H. Buckley, Zijie Feng, Kunfeng Sun, Zhicheng Peng, John W. Tobias, Xianxin Hua, Bryson W. Katona

**Affiliations:** 1 Division of Gastroenterology and Hepatology, Perelman School of Medicine, University of Pennsylvania, Philadelphia, PA 19104, USA; 2 Department of Cancer Biology, Abramson Family Cancer Research Institute, Perelman School of Medicine, University of Pennsylvania, Philadelphia, PA 19104, USAhuax@pennmedicine.upenn.edu (X.H.); 3 Department of Genetics, Penn Genomics Analysis Core, University of Pennsylvania, Philadelphia, PA 19104, USA; jtobias@pennmedicine.upenn.edu

**Keywords:** menin, LXR, colorectal cancer, cholesterol, ABCA1, ABCG1

## Abstract

**Simple Summary:**

Colorectal cancer (CRC) is the leading cause of cancer-related death worldwide, and new therapeutic approaches are direly needed to improve the outcomes of metastatic disease. Herein, we uncover that menin, a nuclear scaffold protein that has a myriad of tissue-specific effects on gene transcription, serves as a novel regulator of cholesterol homeostasis in CRC cell lines in vitro and in the benign colonic epithelium in vivo. Specifically, we demonstrate that menin inhibits the transcription of LXR-regulated genes, including the cholesterol exporters *ABCA1* and *ABCG1*, leading to increased cellular cholesterol content. Conversely, menin inhibition reduces total cellular cholesterol content and sensitizes CRC to small molecule EGFR inhibitors and lipid-poor conditions. These combined findings demonstrate that menin is a key regulator of cholesterol homeostasis in both CRC and the colonic epithelium, and targeting menin may be an effective route for improving therapies for CRC.

**Abstract:**

Background and Aims: Menin is a nuclear scaffold protein that regulates gene transcription in an oftentimes tissue-specific manner. Our previous work showed that menin is over-expressed in colorectal cancer (CRC); however, the full spectrum of menin function in colonic neoplasia remains unclear. Herein, we aimed to uncover novel menin-regulated pathways important for colorectal carcinogenesis. Methods: RNA-Seq analysis identified that menin regulates LXR-target gene expressions in CRC cell lines. Isolated colonic epithelium from *Men1*^f/f^;*Vil1*-Cre and *Men1*^f/f^ mice was used to validate the results in vivo. Cholesterol content was quantified via an enzymatic assay. Results: RNA-Seq analysis in the HT-29 CRC cell line identified that menin inhibition upregulated LXR-target genes, specifically *ABCG1* and *ABCA1*, with protein products that promote cellular cholesterol efflux. Similar results were noted across other CRC cell lines and with different methods of menin inhibition. Consistent with *ABCG1* and *ABCA1* upregulation, and similarly to LXR agonists, menin inhibition reduced the total cellular cholesterol in both HT-29 and HCT-15 cells. To confirm the effects of menin inhibition in vivo, we assessed *Men1*^f/f^;*Vil1*-Cre mice lacking menin expression in the colonic epithelium. *Men1*^f/f^;*Vil1*-Cre mice were found to have no distinct baseline phenotype compared to control *Men1*^f/f^ mice. However, similarly to CRC cell lines, *Men1*^f/f^;*Vil1*-Cre mice showed an upregulation of *Abcg1* and a reduction in total cellular cholesterol. Promoting cholesterol efflux, either via menin inhibition or LXR activation, was found to synergistically suppress CRC cell growth under cholesterol-depleted conditions and when administered concomitantly with small molecule EGFR inhibitors. Conclusions: Menin represses the transcription of LXR-target genes, including *ABCA1* and *ABCG1* in the colonic epithelium and CRC. Menin inhibition conversely upregulates LXR-target genes and reduces total cellular cholesterol, demonstrating that menin inhibition may be an important mechanism for targeting cholesterol-dependent pathways in colorectal carcinogenesis.

## 1. Introduction

Menin is a nuclear scaffold protein encoded via the multiple endocrine neoplasia type 1 (*MEN1*) gene [1,2]. Menin has classically been described as a tumor suppressor, where germline inactivating variants in *MEN1* promote the development of tumors in the pituitary, parathyroid, and endocrine pancreas leading to multiple endocrine neoplasia type 1 (MEN1) syndrome [2,3]. However, menin does not categorically function as a tumor suppressor and instead can serve as a contextual tumor promoter in a highly tissue-specific manner [1]. In mixed lineage leukemia (MLL)-fusion leukemia, menin acts as a tumor promoter by serving as a scaffold to recruit MLL-fusion proteins and facilitate their histone H3 lysine 4 (H3K4) methylation, which subsequently drives leukemogenesis [4]. Menin also acts as a contextual tumor promoter in prostate cancer via the promotion of androgen receptor signaling [5].

Recent evidence has demonstrated that menin may also function as a contextual tumor promoter in colorectal cancer (CRC) [6]. Menin is upregulated in CRC and promotes the transcription of pro-oncogenic SKP2, which was found to be important for mediating resistance to small molecule inhibitors of EGFR (iEGFRs) [6]. Menin also regulates metabolism in CRC cells where it represses glycolysis [7]. Despite the growing evidence that menin serves important functions in CRC, the breadth of menin function in CRC and the colonic epithelium remains incompletely characterized.

CRC is the third most common cause of cancer and cancer-related mortality in both men and women [8]. While 5-year survival in localized CRC is more than 90%, 5-year survival with metastatic disease remains at less than 20%, highlighting that new systemic treatment modalities are still needed for advanced disease [9]. One pathway that may have promise for developing novel therapeutics for CRC is cholesterol homeostasis, as cholesterol dysregulation plays an important role in colorectal carcinogenesis [10,11]. Notably, statins, which are drugs that decrease cholesterol synthesis in the liver via the inhibition of HMG-CoA reductase, have been associated with lower cancer-related mortality in patients with various types of cancer, including CRC [12,13].

One important regulator of cholesterol homeostasis is the liver X receptors (LXRs), including both LXRα and LXRβ, which are transcription factors that function in a sterol-dependent manner [14]. Specifically, after the activation of endogenous ligands such as oxysterols, LXRs regulate the expression of genes involved in the transport and synthesis of cholesterol [15]. This LXR-cholesterol axis has been well described, and LXR activation has been shown to be a potent tumor-suppressing mechanism in brain and breast cancer, among others [16,17]. LXRα’s activation has also been shown to increase the mRNA levels of ABCA1, ABCG5, and ABCG8, which are important transporters for cholesterol efflux. Additionally, the activation of LXRα reduced the growth of CRC cell lines and CRC tumor xenografts and suppressed the development of colonic neoplasia in a mouse model of familial adenomatous polyposis [18]. Although menin has been shown to directly interact with LXRα and inhibit its transcriptional activity in hepatocytes [19], the mechanism of this interaction as well as its relevance to other tissue types remains uncertain.

Herein, we utilize unbiased RNA-Seq to identify that the transcription of LXR-target genes is an important menin-regulated pathway in CRC. We demonstrate that menin serves as a repressor of the transcription of LXR-target genes in both CRC cell lines and benign colonic epithelium in vitro and in vivo. Furthermore, we show that the inhibition of menin reduces total cellular cholesterol in these same models and that menin inhibitors and LXR activators have a similar ability to suppress CRC cell growth when combined with either small molecule EGFR inhibitors or cholesterol-depleted conditions. Taken together, our results illustrate that menin is a novel regulator of cholesterol homeostasis in the colonic epithelium and in CRC. Therefore, targeting menin may be a promising new mechanism for developing new therapies for CRC.

## 2. Materials and Methods

### 2.1. Reagents

Gefitinib (#G-4408) was obtained from LC Laboratories, Woburn, MA, USA, MI-2-2 was obtained from Chemzon Scientific, Quebec, Canada, and MI-463 and MI-503 were obtained from Wuxi Pharma, Shanghai, China. Doxycycline (#D9891) was obtained from Sigma-Aldrich, Burlington, MA, USA. GW3965 was obtained from Tocris Bioscience, Bristol, UK (#2474). T0901317 (#HY-10626) was obtained from Med Chem Express, Monmouth Junction, NJ, USA. Antibodies for PARP (#9542) and GAPDH (#2118) were purchased from Cell Signaling Technology, Danvers, MA, USA. The antibody for actin (#A5441) was purchased from Millipore-Sigma, St. Louis, MO, USA. The antibodies for menin (#A300-105A used for western blot, and #IHC-00572 used for IHC) were purchased from Bethyl Laboratories, Montgomery, TX, USA. Anti-rabbit and anti-mouse secondary antibodies were purchased from Bio-Rad, Hercules, CA, USA. Lipid-depleted FBS was obtained from Omega Scientific, West Lake Village, CA, USA (#FB-50).

### 2.2. Cell Culture

The human colon adenocarcinoma cell lines, HT-29, HCT-15, and HCT-116, were obtained with the Cell Culture Core of the NIH/NIDDK Center for Molecular Studies in Digestive and Liver Diseases at the University of Pennsylvania (Philadelphia, PA, USA). Cell line authentication was performed via short tandem repeat profiling by the ATCC. 293T cells were purchased from the ATCC. All cell lines were used within 10–15 passages of their receipt from the above sources and were regularly tested for *Mycoplasma*. Unless otherwise specified, all cell lines were maintained in DMEM, supplemented with 10% heat-inactivated FBS, 100 U/mL penicillin, and 100 μg/mL streptomycin, and were maintained at 37 °C in a humidified 5% CO_2_ atmosphere.

### 2.3. RNA-Seq

HT-29 cells were plated in 10 cm plates at 2 × 10^6^ cells/plate. After adhering overnight, the cells were treated with DMSO or 1 µM MI-2-2 and were then collected after 30 h. Collected cells were dissolved in Trizol, and RNA was subsequently isolated using an RNeasy Mini Kit (Qiagen, Hilden, Germany) and frozen at −80 °C until the time of analysis. RNA-Seq experiments were performed in triplicate.

RNA-Seq was performed by the High Throughput Sequencing Core at the Children’s Hospital of Philadelphia—Beijing Genomics Institute. Ribosomal RNA-depleted strand-specific RNA libraries were prepared with TruSeq Stranded Total RNA according to manufacturer’s instructions. Unique Dual Indexes Primers were incorporated for multiplexed high-throughput sequencing. The final product was assessed for its size distribution and concentration using BioAnalyzer High Sensitivity DNA Kit (Agilent Technologies, Santa Clara, CA, USA). Briefly, ribosomal depleted RNA was fragmented, then subjected to reverse transcription, end repair and 3′-end adenylation, and adaptor ligation, indexing followed by 10 cycles of PCR amplification then followed by bead purification (Beckman Coulter, Brea, CA, USA). The resulting libraries were pooled, diluted to 2 nM using 10 mM Tris-HCl, pH 8.5, denatured, and loaded onto a PE100 flow cell. Sequencing was performed on a HiSeq2500 Instrument (Illumina, San Diego, CA, USA) with 2 × 100 cycles, using the Illumina TruSeq Rapid SBS sequencing chemistry and following the manufacturer’s instructions. Images from the instrument were processed using the manufacturer’s software to generate FASTQ sequence files. De-multiplexed and adapter-trimmed sequencing reads were generated using Illumina bcl2fastq. Read quality was assessed by running FastQC on the FASTQ files according to the manufacturer’s instructions.

Subsequently, using the FASTQ files on the Penn High-Performance Computer environment, Salmon was used to count the data against the transcriptome defined in Gencode v40, which was built on the genome GRCh38. On a local workstation, several Bioconductor packages in R were used for subsequent steps. The transcriptome count data were annotated and summarized to the gene level with tximeta and further annotated with biomaRt. PCA analysis and plots were generated with PCAtools. Normalizations and statistical analyses were conducted with DESeq2. Exploratory GSEA pathway analysis was conducted with fgsea against the hallmark pathway set from the Molecular Signatures Database (MSigDB), using the DESeq2 statistic as a ranking metric. Heatmap was generated using BioJupies [20] and included only genes with a statistically significant (*p* < 0.05) aggregate 1.5-fold increase or decrease in gene expression between DMSO and MI-2-2 treated samples.

### 2.4. The Cancer Genome Atlas Database Analysis

Level 3 HiSeq RNASeq data were downloaded from The Cancer Genome Atlas (TCGA) for 302 colon samples (40 normal and 262 tumors), and raw counts for each gene in each sample were extracted. Raw counts were imported into R [21], where DESeq2 [22] was applied to score genes for differential expression between tumor and normal samples. For purposes of visualization, DESeq2-calculated normalized log2-transformed counts for each sample, which were then exported.

### 2.5. RT-qPCR

RNA was extracted from cultured cells with Trizol and subsequently isolated using an RNeasy Mini Kit (Qiagen). RNA was transcribed into cDNA, and real-time PCR (RT-qPCR) was performed using a Quantitative SYBR-Green PCR Kit (Qiagen) and a 7500 Fast Real-Time PCR System (Applied Biosystems, Waltham, MA, USA). Sequences of primer sets used are included in Supplemental Table S1. Transcript levels were normalized to either actin or GAPDH, with mean values ± SD reported for each group. All experiments were performed in at least one duplicate.

### 2.6. Plasmids and Transfections

All plasmids were purified utilizing a GenElute HP Plasmid Midiprep Kit (Sigma) after transformation using DH5α cells with ampicillin. Lentiviral packing plasmids, pMD2.G and psPAX2, were purchased from Sigma. LentiCRISPRv2 was obtained as a gift from Dr. Anil Rustgi (Columbia University, New York, NY, USA). The single-guide RNA (sgRNA) sequence targeting the *MEN1* gene (Supplemental Table S1) was cloned into the lentiCRISPRv2 vector as previously described [6]. shRNA plasmids for LXRα were obtained from the University of Pennsylvania Perelman School of Medicine High-Throughput Screening Core (Philadelphia, PA, USA), all of which were derived from a pLKO.1-puromycin backbone, with sequences for the LXRα shRNAs listed in Supplemental Table S1. The pLKO-Tet-On was obtained from Addgene and used to generate doxycycline-inducible menin shRNAs using two validated menin shRNA sequences (Supplemental Table S1) as previously described [23,24]. To produce lentivirus, 293T cells were transfected with pMD2.G, psPAX2, and the plasmid of interest using Fugene 6 (Promega, Madison, WI, USA) according to the manufacturer’s instructions. After collecting and filtering the virus, cells were then transduced in the presence of 4 μg/mL polybrene (hexadimethrine bromide). Twenty-four hours after completion of transduction, cells were then selected with puromycin for 72 h.

### 2.7. Protein Detection via Western Blotting

HT-29 cells were plated in 10 cm plates at a density of 2 × 10^6^ cells/plate, and HCT-15 cells were plated in 10 cm plates at a density of 10^6^ cells/plate. After attaching overnight, the cells were treated as described for the indicated time, followed by collection. For mouse experiments, colonic epithelium was isolated, as described later in the Materials and Methods, and was used for western blotting. Collected cells or colonic epithelium were then lysed with SDS buffer containing protease and phosphatase inhibitors. Protein concentrations were determined using a BCA Assay Kit (Thermo Fisher Scientific). Cell lysates were subjected to PAGE on Novex gels (Life Technologies, Carlsbad, CA, USA), and protein was transferred to polyvinylidene difluoride membranes (Life Technologies). Blocking was performed in TBST containing 5% nonfat dry milk or 5% BSA based on the antibody-blocking instructions recommended by the manufacturer. The proteins were visualized by detection with Amersham ECL Western blotting detection reagents (GE Healthcare).

### 2.8. Chromatin Immunoprecipitation (ChIP) Assay

HT-29 cells were plated at a density of 2 × 10^6^ cells per 10 cm plate and treated for 30 h with two 10 cm plates used for each treatment condition. After the collection of cells, ChIP assays were performed according to the manufacturer’s instructions using a QuickChIP kit (Novus Biologicals, Littleton, CO, USA). Briefly, cells were fixed with 1% formaldehyde for 10 min and then lysed according to the manufacturer’s protocol in a ChIP lysis buffer with protease inhibitors, and cellular DNA was sheared with sonication. This lysate was pre-cleared and then incubated with either control IgG or a specific primary antibody (4 μg) at 4 °C overnight and collected with protein A/G agarose beads. The protein-DNA complexes were eluted from the beads, then DNA was de-crosslinked and amplified via PCR using primer pairs specific to the LXR response element (LXRE) of either *ABCA1* or *ABCG1* (Supplemental Table S1), using a quantitative SYBR-Green PCR Kit (Qiagen) and a 7500 Fast Real-Time PCR System (Applied Biosystems). All reactions were performed in triplicate, and results were normalized to input chromatin and reported as percent input +/− SD.

### 2.9. Mouse Husbandry and Models

All mouse experiments in this study followed NIH Guidelines for the Care and Use of Laboratory Animals and are also in accordance with the IACUC standards following ethics approval by the animal committee at the University of Pennsylvania. *Men1*^fl/fl^ mice were generated as previously described [25]. Villin-Cre (*Vil1*-Cre) mice were obtained from the Jackson Laboratory (B6.Cg-Tg(Vil-Cre)997Gum, JAX stock #: 004586) [26]. *Men1*^fl/fl^ mice and *Vil1*-Cre mice were used to breed *Men1*^fl/fl^;*Vil1*-Cre mice. Genotyping was performed with a REDextract-N-Amp Tissue PCR Kit from Sigma-Aldrich (#P8240) as per the manufacturer’s instructions. Primers used for genotyping are included in Supplemental Table S1.

### 2.10. Mouse Epithelium Isolation

To isolate mouse colonic epithelium, abdominal dissection was performed to remove the mouse colon. The colon was washed using a 5 mL syringe with a bead-tipped needle and splayed using a single, longitudinal cut from the anus to the proximal colon. The colon was then washed 5 times in PBS and transferred to mouse epithelial dissociation buffer composed of 10 mL PBS, 200 µL 1 M EDTA, and 20 µL N-acetyl cystine. After dissociating for 1 h at 4 °C, the epithelium was separated by pipetting the whole colon repeatedly against the bottom of a 50 mL conical using an electric pipette 20 times. The contents of the conical were filtered through a 70 µm filter and spun down at 500× *g* for 5 min to isolate the colonic epithelial pellet.

### 2.11. Cholesterol Quantitation

Adherent cell line pellets were collected by scraping cells, and then spinning down at 1000× *g* for 5 min. Colonic epithelial cell pellets were collected as described above. After supernatant removal, the cells were resuspended in 1 mL of ice-cold PBS. In total, 75% of this cell suspension was used for cholesterol extraction, and 25% was used for a BCA assay. The samples were then spun down again at 1000× *g* for 5 min. The Stanbio Laboratory Enzymatic Cholesterol Liquicolor kit was used to quantify total cellular cholesterol from the cell pellets. Briefly, 100 µL of a 2:1 chloroform/methanol mixture was added to each cell pellet, and cells were broken up via trituration. After incubating at room temperature for 20 min and centrifuging for 5 min at 500× *g* and 4 °C, the upper and lower phases were transferred to a new Eppendorf. Then, 20 µL of 0.85% KCl was added to each tube followed by vortexing for 5 s and then incubation at room temperature for 5 min. Next, the samples were centrifuged again at 500× *g* for 5 min. The upper phase was removed, and the samples were allowed to sit in a fume hood overnight to allow for evaporation of the chloroform–methanol solution. The samples were redissolved the next day in 200 µL of 100% EtOH, vortexed for 10 s, and incubated at 37 °C for 30 min. Standards were created per the kit’s specifications and were plated in duplicate in a 96-well plate, with 3 µL per standard. Samples were plated in triplicate, with 30 µL per sample. Finally, 200 µL of the kit’s cholesterol reagent was added to each sample and the plate was incubated at 37 °C for 30 min. Absorbance of each well was recorded at 500 nm using an ELISA plate reader. Separately, a Pierce BCA Protein Assay kit (Thermo Scientific, Waltham, MA, USA) was used per the manufacturer’s instructions to quantify the total protein concentration of the samples. The cholesterol concentration was subsequently normalized to protein concentration. All cell line experiments were performed in at least triplicate, and mouse experiments were performed with 5 replicates.

### 2.12. Immunohistochemistry

Vector Laboratories DAB staining kit (#SK-4100) was purchased and used according to the manufacturer’s instructions. Mouse colons were dissected, swiss rolled, fixed in 10% formalin at 4 °C for 24 h, transferred to 70% ethanol, and embedded in paraffin. Sections were cut with 5 µm thickness by the Molecular Pathology and Imaging Core of the NIH/NIDDK Center for Molecular Studies in Digestive and Liver Diseases at the University of Pennsylvania (Philadelphia, PA, USA), and then sections were deparaffinized, and antigen retrieval was performed in Tris-EDTA buffer (pH 9) at 100 °C for 15 min. After blocking for 1 h at room temperature using a blocking buffer (5% goat serum, 0.05% Tween-20 in PBS), the sections were incubated with a menin antibody diluted 1:1000 and developed for 3 min and left to rest for 5 min. Samples were stained with hematoxylin for 3 min, and sections were visualized using a Lecia microscope with an ICC50 W digital camera.

### 2.13. Cell Proliferation Assays

For the MTS [3-(4,5-Dimethylthiazol-2-yl)-5-(3-carboxymethoxyphenyl)-2-(4-sulfophenyl)-2H-tetrazolium] assay, HT-29 cells were seeded in a 96-well plate at a density of 10^4^ cells/well. HCT-15 cells were seeded in 96-well plates at a density of 5 × 10^3^ cells/well. After adhering overnight, the cells were then treated as described for the indicated time. The MTS Assay Kit (Promega) was utilized to assess cell growth and was performed according to the manufacturer’s instructions. Absorbance of each well was recorded at 490 nm using an ELISA plate reader, and after subtracting a background reading, these results were normalized to control well readings. Each experiment was performed in triplicate, with mean values ± SD reported for each treatment group. *p*-values were calculated using an unpaired two-tailed *t*-test.

### 2.14. Apoptosis Quantification via Caspase-3/7 Assay

For the caspase-3/7 assay, HT-29 cells were seeded in a 96-well plate at a density of 10^4^ cells/well. HCT-15 cells were seeded in a 96-well white opaque bottom plate at a density of 5 × 10^3^ cells/well. After adhering overnight, the cells were then treated as described for the indicated time. The Apo-ONE Homogeneous Caspase-3/7 assay kit (Promega) was utilized to assess apoptosis and was performed according to the manufacturer’s instructions. Fluorescence of each well was recorded at an excitation wavelength of 499 nm with an emission maximum of 521 nm using an ELISA plate reader, and after subtracting a background reading, these results were normalized to control well readings. Each experiment was performed in triplicate, with mean values ± SD reported for each treatment group. *p*-values were calculated using an unpaired two-tailed *t*-test.

## 3. Results

### 3.1. Menin Inhibition Increases LXR Target Gene Expression in CRC Cells 

**Figure 1:**
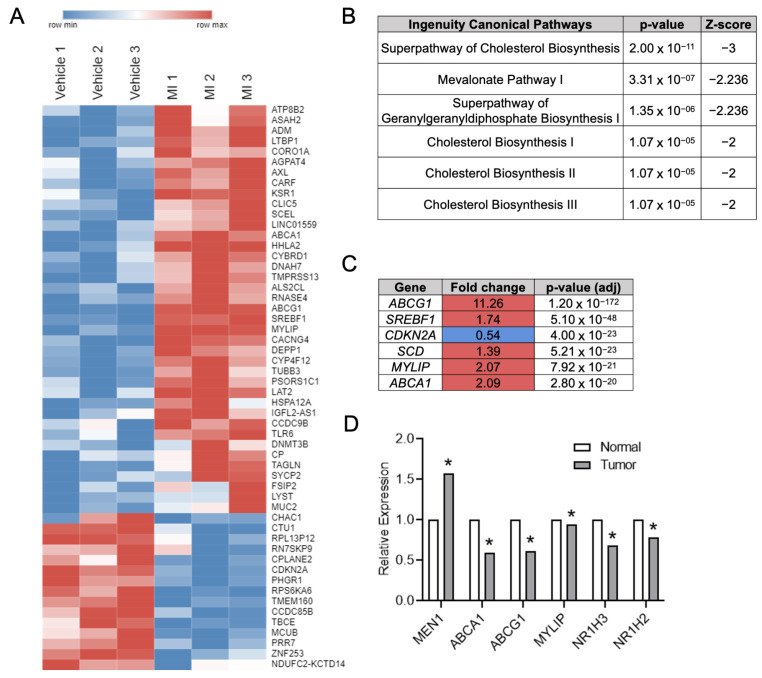
Menin inhibition in CRC cells leads to upregulation of LXR target genes. (**A**) RNA-Seq performed on HT-29 cells treated with either vehicle or 1 µM MI-2-2 for 30 h. Genes with a net log2-fold change ≥0.5 and *p*-value < 0.05 are displayed in a heatmap. Red coloring represents upregulation, and blue coloring represents downregulation. (**B**) Top regulated pathways after Ingenuity Pathway Analysis of RNA-Seq data from HT-29 cells. (**C**) Genes with the lowest *p*-values from HT-29 RNA-Seq data. Red coloring represent upregulation after MI-2-2 treatment, and blue coloring represents downregulation. (**D**) Relative expression differences between benign colonic epithelium and CRC from the TCGA. * *p* < 0.05.

To assess for novel menin-regulated targets in CRC, RNA-Seq was performed in HT-29 CRC cells after treatment with the small molecule menin inhibitor (MI) MI-2-2 (Figure 1A). Ingenuity pathway analysis demonstrated significant pathway alterations after menin inhibition in cholesterol-related pathways (Figure 1B). Furthermore, of the six genes with the smallest *p*-values after MI treatment, the majority were LXR transcriptional targets involved in cholesterol homeostasis (*ABCG1*, *SREBF1*, *MYLIP*, and *ABCA1*) (Figure 1C). The analysis of CRCs from The Cancer Genome Atlas (TCGA) demonstrated an inverse relationship between menin expression and LXRα (*NR1H3*) and LXRβ (*NR1H2*) expression, as well as LXR-target gene (*ABCA1*, *ABCG1*, and *MYLIP*) expression (Figure 1D).

**Figure 2:**
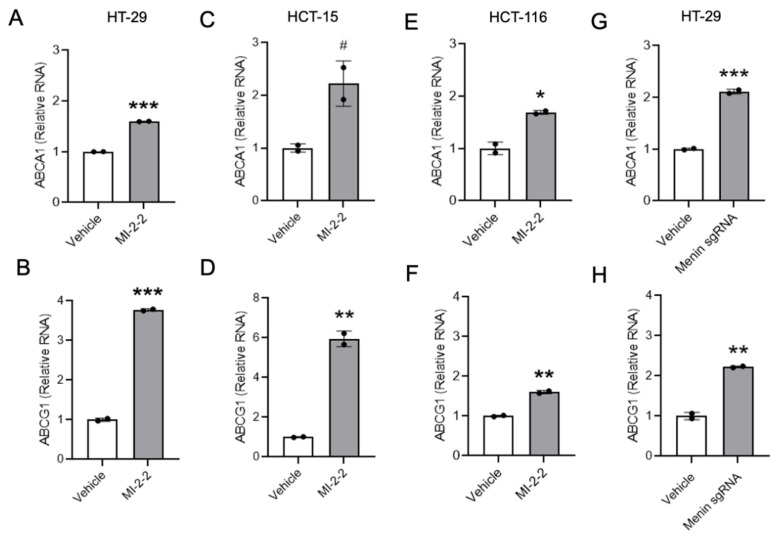
Menin inhibition increases expression of LXR target genes in CRC cells. (**A**–**F**) HT-29 cells (**A**,**B**), HCT-15 cells (**C**,**D**), or HCT-116 cells (**E**,**F**) were treated with vehicle (DMSO) or 1 μM MI-2-2 for 96 h, and expression of ABCA1 and ABCG1 was analyzed using RT-qPCR relative to actin. (**G**,**H**) HT-29 cells were treated with empty vector (vehicle) or menin-directed sgRNA for 96 h, and expression of ABCA1 (**G**) and ABCG1 (**H**) was analyzed using RT-qPCR relative to actin. * *p* < 0.05. ** *p* < 0.01. *** *p* < 0.001. # *p* < 0.06.

Similar effects of menin inhibition on the LXR-target gene expression of the cholesterol transporters ABCA1 and ABCG1 were observed using RT-qPCR after the treatment of HT-29 cells with MI-2-2 (Figure 2A,B) as well as other small molecule menin inhibitors MI-503 and MI-463 (Supplementary Figure S1A,B) [27]. The increase in LXR-target gene expression was also noted across other CRC cell lines, including HCT-15 (Figure 2C,D) and HCT-116 (Figure 2E,F) cells. As off-target effects of small molecule MIs are possible [28], menin expression was reduced in HT-29 cells with both CRISPR/Cas9 targeting *MEN1* (Figure 2G,H, Supplementary Figure S1C) and doxycycline-inducible menin shRNAs (Supplementary Figure S1D–G), leading to the increased expression of both ABCA1 and ABCG1. Together, these data illustrate that menin inhibition increases the expression of LXR-target genes.

### 3.2. Menin Localizes to LXR-Target Gene Promoters and Suppresses Transcription

**Figure 3:**
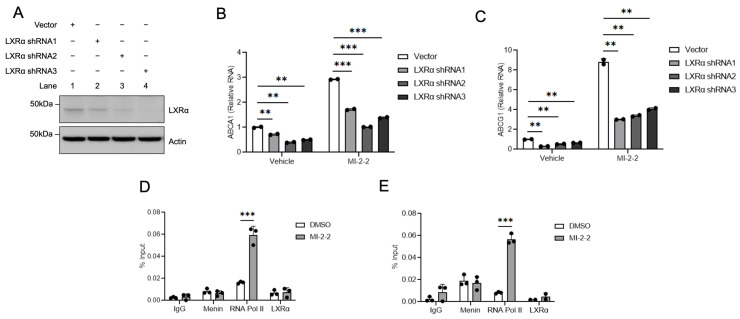
Menin inhibition increases active gene transcription at LXR target gene promoters. (**A**) HT-29 cells were treated with either vector or an LXRα shRNA, and protein levels were assessed via western blot. (**B**,**C**) HT-29 cells were transduced with either vector or an LXRα shRNA and then treated with vehicle or 1 µM MI-2-2 for 72 h, followed by assessment of ABCA1 (**B**) and ABCG1 (**C**) expression relative to actin using RT-qPCR. (**D**,**E**) ChIP assay was performed at an LXRE at the promoter of *ABCA1* (**D**) and *ABCG1* (**E**) in HT-29 cells after treatment with vehicle or 1 μM MI-2-2 for 48 h. ** *p* < 0.01. *** *p* < 0.001.

To determine if menin-mediated changes in LXR-target gene expression are LXR-dependent, LXRα was knocked down with shRNAs (Figure 3A) in HT-29 cells leading to a reduction in ABCA1 (Figure 3B) and ABCG1 (Figure 3C) expression at baseline and after MI-2-2 treatment. Using ChIP assays, menin was found to localize to the LXR response elements (LXREs) of the promoters of the LXR-target genes *ABCA1* and *ABCG1* (Figure 3D,E). While menin inhibition with MI-2-2 did not reduce the level of menin at the LXREs, this inhibition did lead to increased RNA polymerase II at the LXREs of both *ABCA1* and *ABCG1*, consistent with increased transcriptional activity without increased recruitment of LXRα (Figure 3D,E). These results demonstrate that menin localizes to the LXREs of LXR-target genes and represses transcription.

### 3.3. Menin Inhibition Decreases Cellular Cholesterol

**Figure 4:**
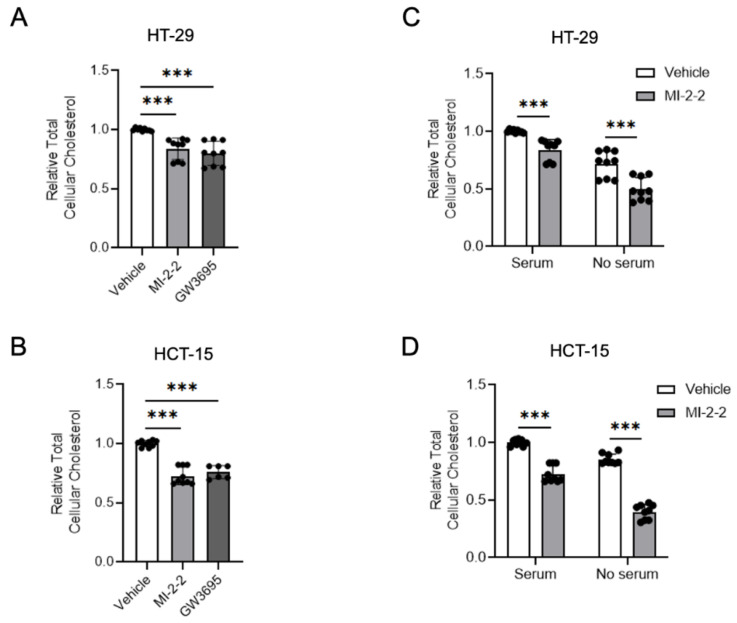
Menin inhibition decreases cellular cholesterol. (**A**,**B**) HT-29 (**A**) and HCT-15 (**B**) cells were treated with 1 µM MI-2-2 and 5 µM GW3965 for 48 h; total cellular cholesterol was quantified and normalized to protein concentration. (**C**,**D**) HT-29 (**C**) and HCT-15 (**D**) cells were treated with 1 µM MI-2-2 and 5 µM GW3965 in media containing 10% FBS or serum-free media for 48 h; total cellular cholesterol was quantified and normalized to protein concentration. *** *p* < 0.001.

As menin inhibition increased LXR-target gene expressions, including the cholesterol exporters ABCA1 and ABCG1, we next investigated whether these transcriptional changes led to detectable alterations in cellular cholesterol homeostasis. The treatment of HT-29 cells with either MI-2-2 or the LXR agonist GW3965 [29] led to reductions in total cellular cholesterol compared to vehicle-treated cells (Figure 4A). Similar effects were observed in HCT-15 cells (Figure 4B). Reductions in total cellular cholesterol were further magnified in both HT-29 (Figure 4C) and HCT-15 cells (Figure 4D) when cultured in serum-free (i.e., cholesterol-poor) conditions. These data demonstrate that menin’s effects on LXR target genes have an observable effect on total cellular cholesterol levels.

### 3.4. Loss of Menin Decreases Colonic Epithelial Cholesterol in Mice

**Figure 5:**
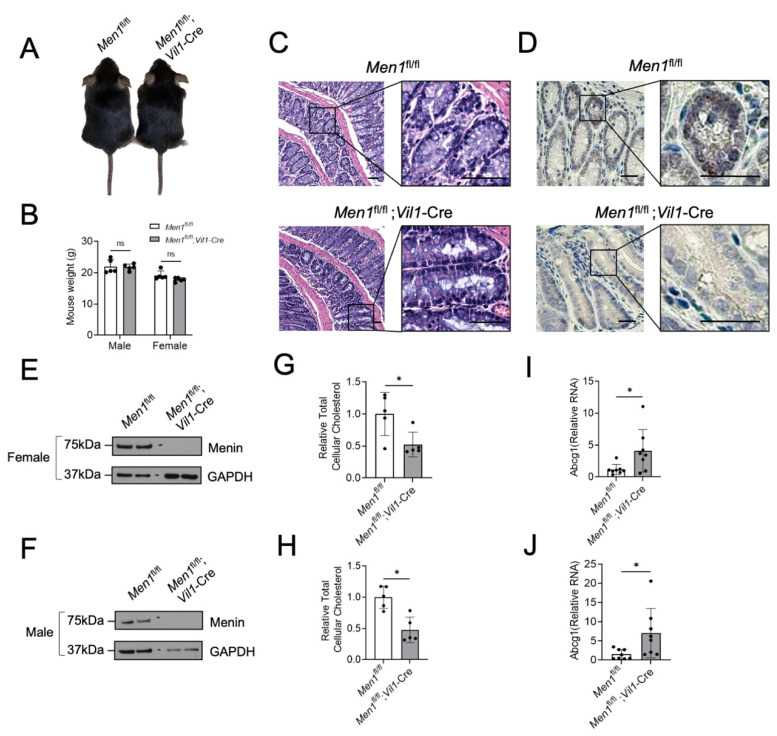
Targeted deletion of the *Men1* gene in mouse colonic epithelium reduces colonic epithelial cholesterol content. (**A**) Representative picture of a *Men1*^fl/fl^ mouse and *Men1*^fl/fl^;*Vil1*-Cre mouse. (**B**) Weight in grams of male and female *Men1*^fl/fl^ and *Men1*^fl/fl^;*Vil1*-Cre mice at 8 weeks of age. (**C**) Representative H&E-stained sections of colonic epithelium from 8-week-old *Men1*^fl/fl^ and *Men1*^fl/fl^;*Vil1*-Cre mice, scale bars = 50 µm. (**D**) Representative IHC staining for menin in the colonic epithelium of *Men1*^fl/fl^ mice and *Men1*^fl/fl^;*Vil1*-Cre mice, scale bars = 25 µm. (**E**,**F**) Menin protein assessment using western blot from isolated colonic epithelium of female (**E**) and male (**F**) mice. (**G**,**H**) Relative total cellular cholesterol in isolated mouse colonic epithelium either with or without menin expression in female (**G**) and male (**H**) mice. (**I**,**J**) Abcg1 expression in isolated mouse colonic epithelium with and without menin in female (**I**) and male (**J**) mice assessed via RT-qPCR relative to GAPDH. * *p* < 0.05. ns = not significant (*p* > 0.05).

To determine if menin regulates colonic epithelial cholesterol content in vivo, *Men1*^fl/fl^ and *Men1*^fl/fl^;*Vil1*-Cre mice were generated and used. Importantly, both genotypes appear phenotypically identical, have identical weights, and have normal-appearing colonic epithelial histology (Figure 5A–C). Under the direction of the *Vil1* promoter, *Men1*^fl/fl^;*Vil1*-Cre mice lack menin expression in the colonic epithelium compared to *Men1*^fl/fl^ mice as assessed using menin IHC (Figure 5D) and using both Western blot (Figure 5E,F) and RT-qPCR (Supplementary Figure S2A,B) of isolated colonic epithelium in both female and male mice. Similar to results from CRC cell lines, total cellular cholesterol was reduced in the colonic epithelium of *Men1*^fl/fl^;*Vil1*-Cre mice compared to *Men1*^fl/fl^ mice (Figure 5G,H). Additionally, and also similarly to CRC cell lines, the transcription of the LXR-target gene *Abcg1* was increased in *Men1*^fl/fl^;*Vil1*-Cre mice compared to *Men1*^fl/fl^ mice (Figure 5I,J). Together, these in vivo data demonstrate that the loss of menin reduces cellular cholesterol content in both benign colonic epithelium and CRC.

### 3.5. Menin Inhibition Increases CRC Cell Killing under Cholesterol Depleted Conditions

**Figure 6:**
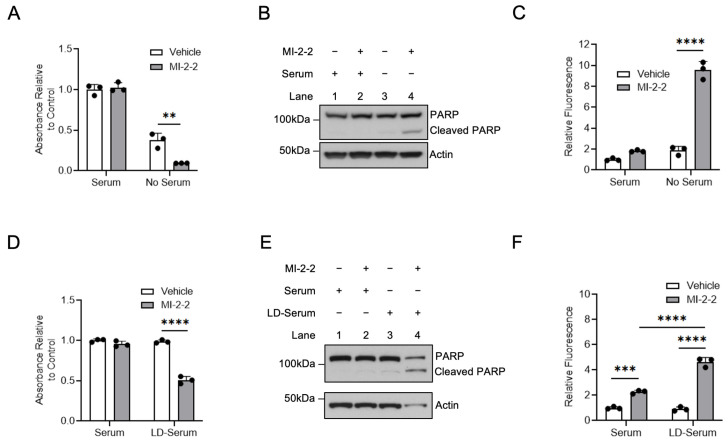
Menin inhibition enhances CRC cell death under lipid-poor conditions. (**A**,**B**) HT-29 cells were treated with 1 µM MI-2-2 for 96 h in media containing 10% FBS (serum) or serum-free media, with cell growth assessed via MTS assay (**A**) and protein assessed via western blot (**B**). (**C**) HT-29 cells were treated with 1 µM MI-2-2 for 72 h in media containing 10% FBS (serum) or serum-free media with apoptosis quantified with caspases-3/7 activity. (**D**–**F**) HT-29 cells were treated with 1 µM MI-2-2 for 96 h in media containing 10% FBS (serum) or 10% lipid-depleted FBS (LD-serum), with cell growth assessed via MTS assay (**D**), protein assessed via western blot (**E**), and apoptosis quantified with caspases-3/7 activity (**F**). ** *p* < 0.01. *** *p* < 0.001. **** *p* < 0.0001.

Maintaining adequate cellular cholesterol levels is important for highly proliferative cancer cells [10,11]; therefore, targeting cholesterol maintenance pathways may be an effective anti-cancer strategy. HT-29 cells treated with MI-2-2 in cholesterol-free (i.e., serum-free) conditions showed a decrease in cell growth (Figure 6A) and increased apoptosis as assessed using both PARP cleavage and caspase-3/7 cleavage (Figure 6B,C) compared to vehicle-treated cells. The treatment of HCT-15 cells with MI-2-2 in serum-free conditions also showed reduced cell growth and increased apoptosis (Supplementary Figure S3A–C). Similar effects were observed with the use of MI-463 and MI-503 in both HT-29 and HCT-15 cells (Supplementary Figure S4A–F). Furthermore, the use of the LXR agonists GW3965 and T0901317 [30] produced results similar to those produced from menin inhibition in HT-29 and HCT-15 cells (Supplementary Figure S5A–H). To confirm that the effects observed with serum-free conditions were, in fact, cholesterol dependent, cells were then treated with menin inhibitors under normal serum or lipid-depleted fetal bovine serum (LD-Serum) conditions. In both HT-29 cells (Figure 6D–F) and HCT-15 cells (Supplementary Figure S3D–F), there was decreased cell growth and increased apoptosis with menin inhibition under lipid-depleted conditions. These results demonstrate that the use of an LXR agonist or menin inhibitor increased CRC cell killing under cholesterol-depleted conditions.

### 3.6. LXR Agonists Synergize with iEGFRs to Kill CRC Cells Similar to Menin Inhibitors

**Figure 7:**
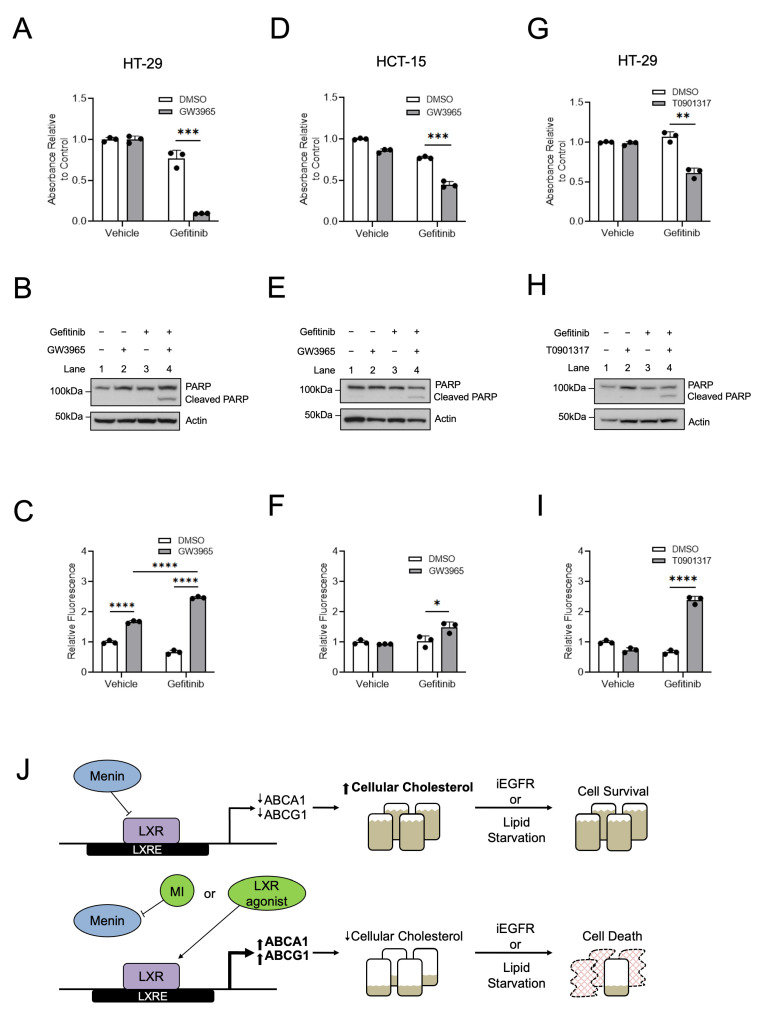
Similar to menin inhibitors, LXR agonists synergize with iEGFRs to suppress CRC. (**A**–**C**) HT-29 cells were treated with 10 µM GW3965 and/or 10 µM gefitinib for 96 h, with assessment of cell growth via MTS assay (**A**) and apoptosis via both PARP cleavage (**B**) and via caspases-3/7 activity (**C**). (**D**–**F**) HCT-15 cells were treated with 10 µM GW3965 and/or 7.5 µM gefitinib for 96 h with assessment of cell growth via MTS assay (**D**) and apoptosis via both PARP cleavage (**E**) and via caspases-3/7 activity (**F**). (**G**–**I**) HT-29 cells were treated with 10 µM T0901317 and/or 10 µM gefitinib for 96 h, with assessment of cell growth via MTS assay (**G**) and apoptosis via both PARP cleavage (**H**) and via caspases-3/7 activity (**I**). (**J**) Proposed model for menin repression of LXR-mediated transcription in CRC. * *p* < 0.05. ** *p* < 0.01. *** *p* < 0.001. **** *p* < 0.0001.

Our previous work demonstrated that MIs synergize with iEGFRs to suppress CRC [6,7]. Given the similarities observed between MIs and LXR agonists, and since the combination of an LXR agonist and iEGFR has not been evaluated in CRC, CRC cell lines were treated with a combined LXR agonist and iEGFR. In HT-29 cells and HCT-15 cells, combined treatment with GW3965 and the iEGFR gefitinib led to decreased cell growth and increased apoptosis (Figure 7A–F). Similarly, the use of the LXR agonist T0901317 also led to decreased cell growth and increased apoptosis when combined with gefitinib (Figure 7G–I). These results indicate that LXR agonists do indeed synergize with iEGFRs to suppress CRC cell growth in vitro in a manner similar to MIs [6,7].

## 4. Discussion

CRC remains one of the most common cancers and causes of cancer-related death despite decades of extensive research [8]. As such, uncovering targetable pathways to improve treatments for CRC is of paramount importance. We have previously shown that menin plays an important role in CRC [6,7]. In this study, we expand the role of menin in CRC by utilizing unbiased approaches to identify both LXR-target genes and cholesterol homeostasis as new menin-regulated pathways in CRC. Our results are notable for multiple reasons. First, we showed that menin binds to LXREs and inhibits the transcription of LXR-target genes. Second, we demonstrated that menin is critical for maintaining cellular cholesterol content in CRC and in the benign colonic epithelium in both in vitro and in vivo settings. Third, we show that the maintenance of cellular cholesterol via menin-mediated LXR inhibition is important for mediating the resistance of CRC to cholesterol-depleted conditions as well as to iEGFRs. Taken together, these findings demonstrate that menin plays an important role in cholesterol homeostasis via the inhibition of LXR-target gene transcription and that targeting menin may serve as a potential mechanism to improve the response of CRC to targeted therapies (Figure 7J).

Cholesterol is an important component of the cellular membrane [31] and maintaining sufficient cellular levels of cholesterol is critical for cancer cell viability given their proclivity for rapid cell division [10,11]. Therefore, defining pathways that regulate cholesterol homeostasis could have a significant impact on developing future cancer therapies [32]. Using unbiased RNA-Seq and Ingenuity pathway analysis, we identified that cholesterol homeostasis and LXR-target gene transcription were pathways significantly altered by a small molecule menin inhibitor in a CRC cell line. Importantly, to date, there has been no report that menin modulates cholesterol content in CRC or the colonic epithelium. We showed that the inhibition of menin, with either small molecule MIs or genetic knockdown, led to increases in LXR-target gene expression, including the cholesterol exporters *ABCG1* and *ABCA1*, in multiple CRC cell lines. Interestingly, menin inhibition also led to a reduction in the total cellular cholesterol across multiple different CRC cell lines. Similar results were observed in vivo, where mice lacking colonic epithelial menin expression showed increased transcription of the LXR-target gene *Abcg1* as well as a significant reduction in total cellular cholesterol. These findings demonstrate that menin’s regulation of cholesterol homeostasis is a physiologically relevant process in the colonic epithelium in vivo.

To begin to understand how menin inhibits the transcription of LXR-target genes, we first utilized a ChIP assay to demonstrate that menin localizes to the LXREs of the promotors for both *ABCG1* and *ABCA1*. Furthermore, treatment with a MI led to increased transcriptional activity at these LXR-target genes; however, this treatment precipitated no change in menin localization at the promoter. It is possible that menin itself acts as a direct repressor of the LXR-mediated transcription or that menin recruits a separate LXR repressor, with both processes potentially being inhibited by an MI or genetic knockdown of menin. Preliminary work in hepatic cells also demonstrated that menin represses LXRα [19]. These data alluded to a potential direct interaction between menin and LXRα, although the specific mechanism was not elucidated [19]. The mechanism whereby menin represses the LXR-mediated gene transcription in CRC and the colonic epithelium will be an area of future investigation.

Menin plays a role in a myriad of cellular processes, often in a tissue-specific manner [1]. Its role in cancer is particularly noteworthy, as menin serves as a tumor suppressor in neuroendocrine cancers [2,3] but can act as a contextual tumor promoter in prostate cancer, MLL-fusion leukemia, and now CRC [4,5]. In this study, we have demonstrated that menin inhibits the transcription of LXR-target genes in CRC as well as in benign colonic epithelium. An interesting area for future study would be to investigate whether this effect is specific to the colon or whether it is also observed more globally. Additionally, statins, which are HMG-CoA reductase inhibitors that lower total body cholesterol, have been associated with lower cancer-related mortality in patients with various types of cancer, including CRC [12,13]. Furthermore, there has been some evidence of the anti-neoplastic effects of statins in CRC. For example, atorvastatin can induce apoptosis and slow the growth of CRC tumor xenografts in vivo [33]. However, statins are currently not regularly utilized as part of CRC treatment regimens. While statins lower available cholesterol for CRC, in contrast, menin inhibition leads to active transcriptional changes that reduce cellular cholesterol. Therefore, future studies of combined menin inhibition along with statin treatment are indicated to determine if both of these cholesterol-depriving inhibitors, working via different mechanisms, may have synergistic effects on CRC.

Apart from CRC, cholesterol homeostasis may also have importance in other pathologic conditions of the colon, such as inflammatory bowel disease (IBD). Recent work has highlighted that the LXR-ABCA1 pathway may demonstrate a protective, anti-inflammatory effect in IBD [34]. Furthermore, prior work has also demonstrated that the loss of LXRβ leads to an increased susceptibility to colitis in mouse models, where treatment with LXR agonists improves recovery from inflammation [35]. As such, it is possible that MIs may also have a role in protecting against inflammation in the colon via their de-repression of LXR-mediated gene transcription and may therefore serve as a potential treatment option for IBD. Further assessment of MIs in mouse models of IBD is certainly warranted to explore this concept.

Targeting cholesterol homeostasis as a potential cancer therapeutic pathway has been examined using multiple LXR agonists [36]. Given the need for rapidly proliferating cancer cells to maintain a robust supply of cholesterol, this approach has had success in laboratory models [37,38]. However, the translation of LXR agonists into clinical use in patients has not been straightforward. Due to the role of LXR gene regulation in multiple tissues, many of these LXR agonists have been associated with adverse effects that have limited their use, including increasing plasma triglycerides and fatty deposition in the liver [39,40,41]. Since menin inhibition has a similar effect on total cellular cholesterol levels compared to LXR agonists, yet likely acts via a different mechanism than direct LXR agonists, it is possible that MIs could have anti-cancer effects with fewer adverse effects. Excitingly, early-phase clinical trials of MIs are currently being conducted in humans, including NCT05631574, NCT05153330, NCT05360160, NCT04067336, NCT04811560, and NCT05731947. Patient data from ongoing early-phase clinical trials of small molecule MIs will be important for understanding the global effect of MIs on patients, including any potentially limiting adverse events, and may allow for an easier transition of MIs to CRC-focused clinical trials.

## 5. Conclusions

In this study, we demonstrate that menin represses LXR-target gene expression in the colonic epithelium and CRC, which helps maintain total cellular cholesterol and protect against metabolic stress such as cholesterol-depleted conditions. These newly identified menin-regulated pathways in CRC may serve as a mechanism to improve future treatments for CRC.

## Data Availability

The data presented in this study are available in this article.

## Supplementary Materials

The following supporting information can be downloaded at: https://www.mdpi.com/article/10.3390/cancers15164126/s1.
