# Supplementary material for: Menin Maintains Cholesterol Content in Colorectal Cancer via Repression of LXR-Mediated Transcription
Source: Cancers (Basel). 2023 Aug 16;15(16):4126. doi: 10.3390/cancers15164126 (PMC10453013; doi:10.3390/cancers15164126)
Supplement: Supplementary file 1 [file cancers-15-04126-s001.zip › cancers-2399358-supplementary-need revise/cancers-2399358 -western blot .pdf]

Main figures:
*Dotted line indicates where membrane was cut for development at different exposure times

Figure 3.A

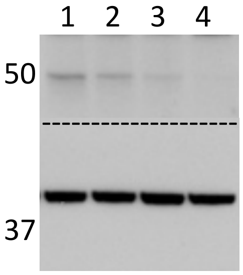

LXRa 100: 54 : 17 : 4
Actin 100:100:107:96

1=vector
2=LXRa shRNA1
3=LXRa shRNA2
4=LXRa shRNA3

Figure 5.E (males)

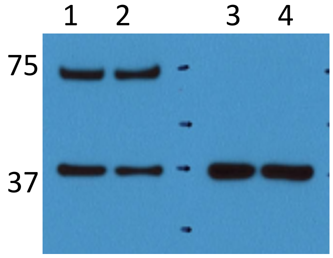

menin	100:100	0 : 0
GAPDH	100:78	146:149

Figure 5.F (females)

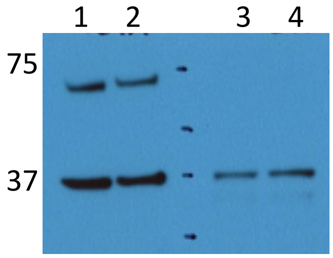

menin	100:81	0 : 0
GAPDH	100:92	30 : 45

1=Menfl/fl
2=Menfl/fl
3=Menfl/fl;Vil1-Cre
4=Menfl/fl;Vil1-Cre

Figure 6.B

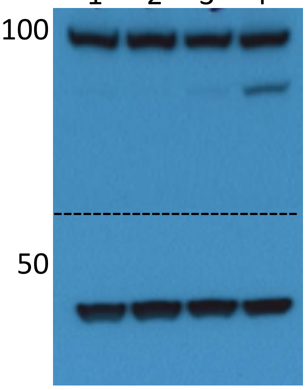

PARP 100:104: 94: 99
Cleaved PARP 100:65:287:3690
Actin 100:114:106:102

1=Serum control
2=Serum MI-2-2
3=No serum control
4=No serum MI-2-2

Figure 6.E

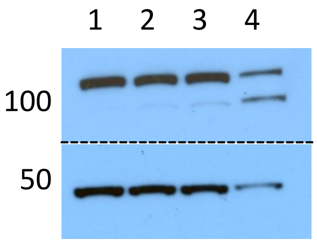

PARP	100:94:94:94
Cleaved	100:160:286:1497
PARP	100:93:85:29

1=Serum control
2=Serum MI-2-2
3=LD-Serum control
4=LD-Serum MI-2-2

Figure 7.B

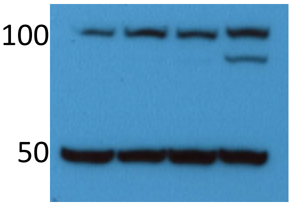

PARP 100:180:165:195
Cleaved PARP 100:85:234:8237
Actin 100:101:117:107

1=Control
2=GW3695
3=Gefitinib
4=Gefitinib + GW3695

Figure 7.E

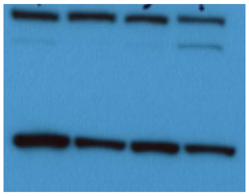

PARP	100:	108:	82:	61
Cleaved PARP	100:	28:	52:	621
Actin	100:	62:	71:	59

1=Control
2=GW3695
3=Gefitinib
4=Gefitinib + GW3695

Figure 7.H

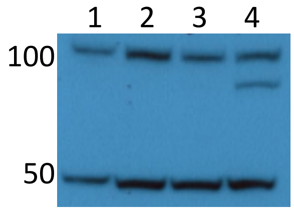

PARP 100:235:147:145
Cleaved PARP 100:36:137:9635
Actin 100:187:178:176

1=Control
2=T0901317
3=Gefitinib
4=Gefitinib + T0901317

Supplementary Figures:

Sup Figure 1.C

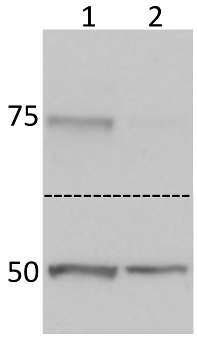

Menin	100:	6
Actin	100:	64

1=Vector
2=Menin sgRNA

Sup figure 1.D

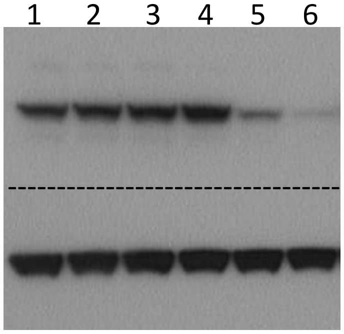

Menin 100:114:130:141:46:13

Actin 100:105:109:110:107:107

1=ind-Scram

2=ind-Menin sgRNA 1

3=ind-Menin sgRNA 2

4=doxy + ind-Scram

5=doxy + ind-Menin sgRNA 1

6=doxy + ind-Menin sgRNA 2

Supplementary Figure 3.B

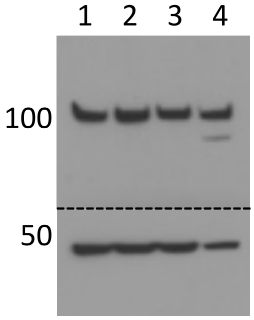

PARP 100:108:92:76
Cleaved PARP 100:124:246:56730
Actin 100:98:89:57

1=Serum control
2=Serum MI-2-2
3=No serum control
4=No serum MI-2-2

Supplementary Figure 3.E

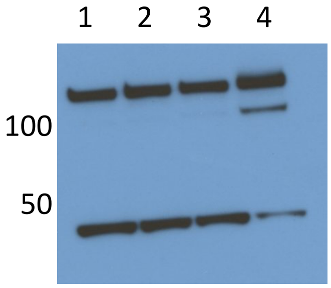

PARP 100:94:66:94
Cleaved PARP 100:135:134:3152
Actin 100:94:94:10

1=Serum control
2=Serum MI-2-2
3=LD-Serum control
4=LD-Serum MI-2-2

Sup figure 4.C

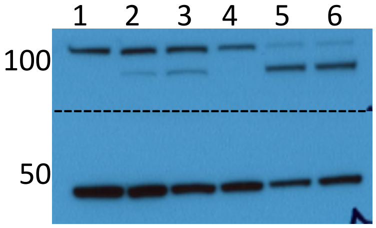

PARP 100:101:104:58:11:8

Cleaved PARP 100:3964:9161:201:32631:34660

Actin 100:95:75:70:42:56

1=serum + Control

2=serum + MI-463

3=serum + MI-503

4=no serum + Control

5=no serum + MI-463

6=no serum + MI-503

Sup figure 4.F

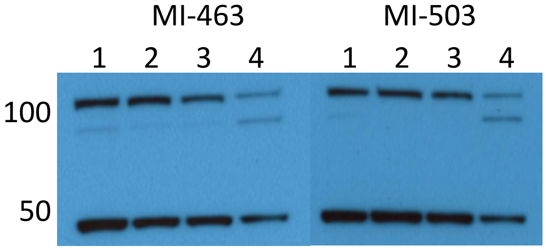

	MI-463	MI-503
PARP	100:97:60:21	100:111:83:24
Cleaved PARP	100:33:25:195	100:21:19:1093
Actin	100:78:71:47	100:104:88:50

1=Serum + control
2=Serum + MI-463
3=No serum + Control
4=No serum + MI-463

Sup figure 5.B

1 2 3 4

100

50

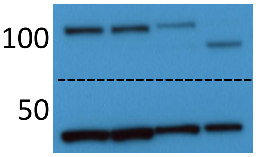

PARP 100:91:32:2

Cleaved PARP 100:54:447:13177

Actin 100:108:73:59

1=Serum + Control

2=Serum + GW3965

3=No serum + Control

4=No serum + GW3965

Sup figure 5.F

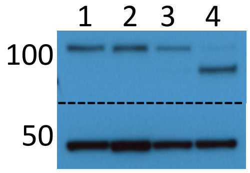

PARP 100:104:48:6
Cleaved PARP 100:116:989:53549
Actin 100:121:92:87

1=Serum + Control
2=Serum + T0901317
3=No serum + Control
4=No serum + T0901317

Sup figure 5.D

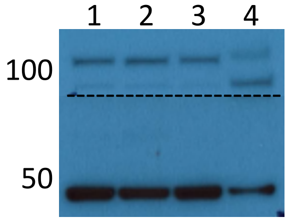

PARP 100:111:90:45
Cleaved PARP 100:96:73:840
Actin 100:81:93:55

1=Serum + Control
2=Serum + GW3965
3=No serum + Control
4=No serum + GW3965

Sup figure 5.H

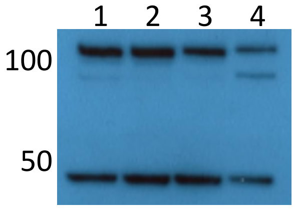

PARP 100:108:86:38
Cleaved PARP 100:29:34:614
Actin 100:127:125:73

1=Serum + Control
2=Serum + T0901317
3=No serum + Control
4=No serum + T0901317
