# Supplementary material for: Menin Maintains Cholesterol Content in Colorectal Cancer via Repression of LXR-Mediated Transcription
Source: Cancers (Basel). 2023 Aug 16;15(16):4126. doi: 10.3390/cancers15164126 (PMC10453013; doi:10.3390/cancers15164126)
Supplement: Supplementary file 1 [file cancers-15-04126-s001.zip › cancers-2399358-supplementary-need revise/cancers-2399358-Supp Final fix.pdf]

Supplementary Data

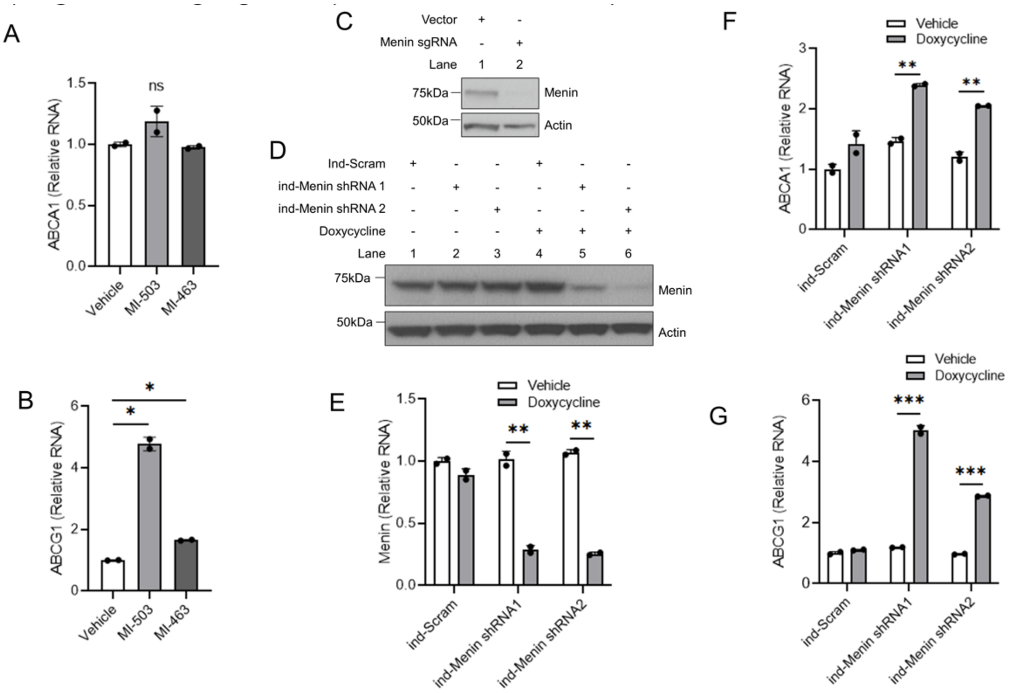

Supplementary Figure S1. LXR target gene expression is increased by menin inhibition. A/B) HT-29 cells were treated with 1 μM MI-503 and 1 μM MI-463 for 72 hours and expression of ABCA1 (A) and ABCG1 (B) was measured by RT-qPCR relative to actin. C) HT-29 cells were treated with vector or menin directed sgRNA for 96 hours, and protein levels were assessed by western blot. D) HT-29 cells were treated with vector or a doxy-inducible shRNA, followed by treatment with 200 ng/mL of doxycycline for 24 hours, and protein levels were measured by western blot 48 hours later. E/F/G) HT-29 cells were treated with vector or a doxy-inducible shRNA, followed by treatment with 200 ng/mL of doxycycline for 24 hours, and RNA expression levels of menin (E), ABCA1 (F), ABCG1 (G) were measured by RT-qPCR relative to actin after 48 hours. * $p < 0.05$. ** $p < 0.01$. *** $p < 0.001$. ns = not statistically significant ($p > 0.05$)

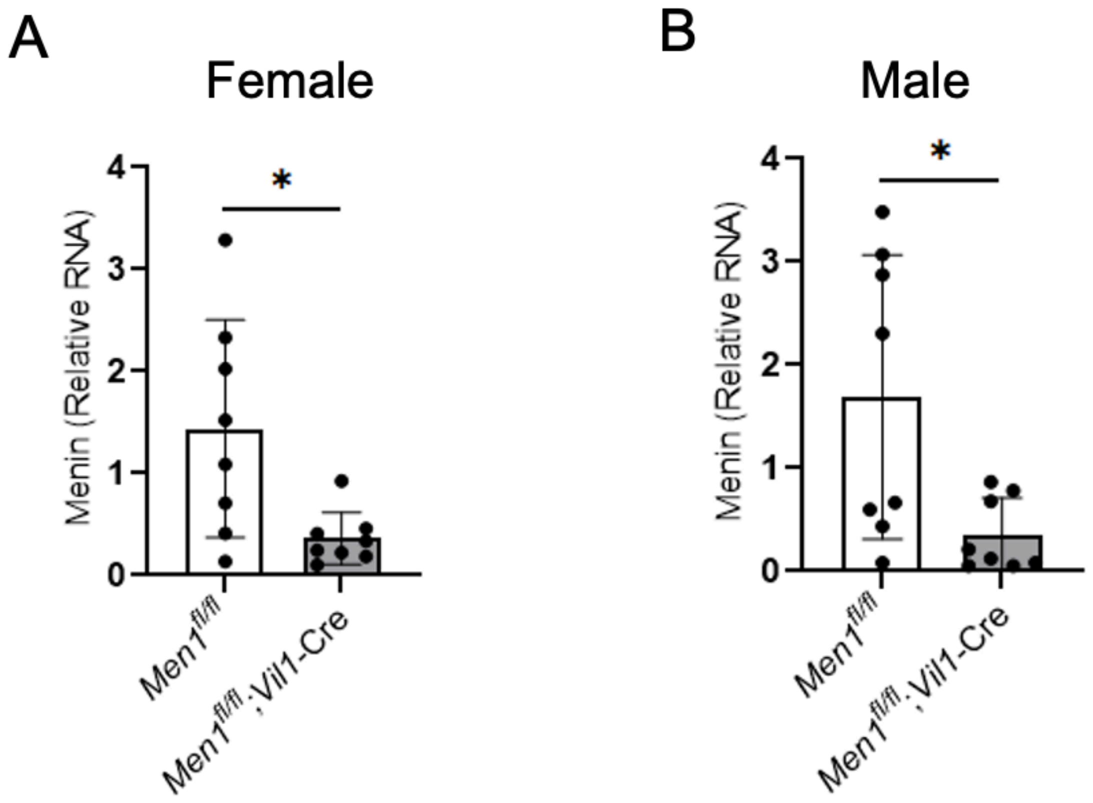

Supplementary Figure S2. Colonic epithelial menin RNA levels in mice. A/B) Menin expression in isolated colonic epithelium in female (A) and male (B) mice assessed by RT-qPCR relative to GAPDH. * $p < 0.05$

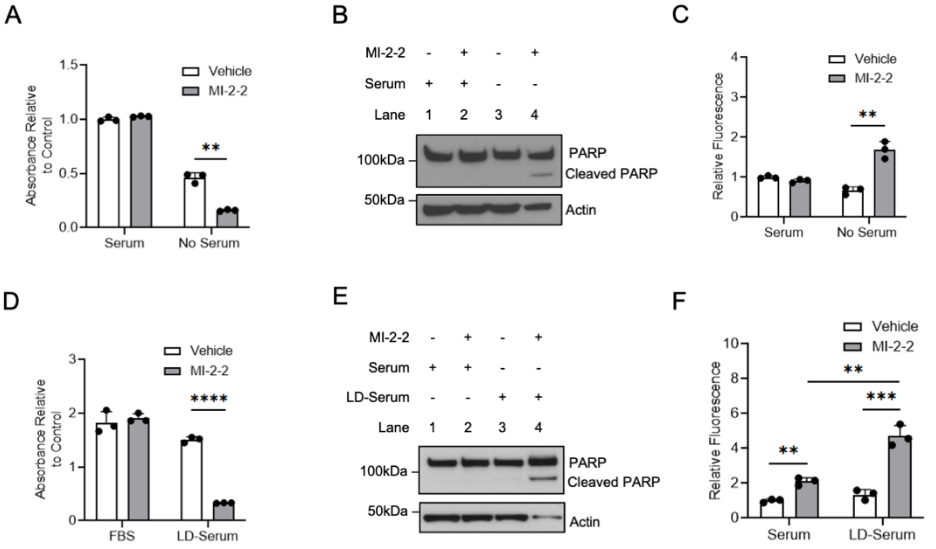

Supplementary Figure S3. Menin inhibition enhances cell death of CRC cells under lipid poor conditions. (A–C) HCT-15 cells were treated with 1 μM MI-2-2 for 96 hours in serum containing (10% FBS) or serum-free media, with cell growth assessed by MTS assay (A), protein assessed by western blot (B), and apoptosis quantified by caspase-3/7 activity (C). D) HCT-15 cells were treated with 1 μM MI-2-2 for 96 hours in media contining 10% FBS (serum) or 10% lipid-depleted FBS (LD-FBS), with cell growth assessed by MTS assay. E) HCT-15 cells were treated with 1 μM MI-2-2 for 72 hours in media contining 10% FBS (serum) or 10% lipid-depleted FBS (LD-FBS), with protein assessed by western blot. F) HCT-15 cells were treated with 1 μM MI-2-2 for 96 hours in media contining 10% FBS (serum) or 10% lipid-depleted FBS (LD-FBS), with apoptosis quantified by measuring caspase-3/7 activity. ** $p < 0.01$. *** $p < 0.001$. **** $p < 0.0001$.

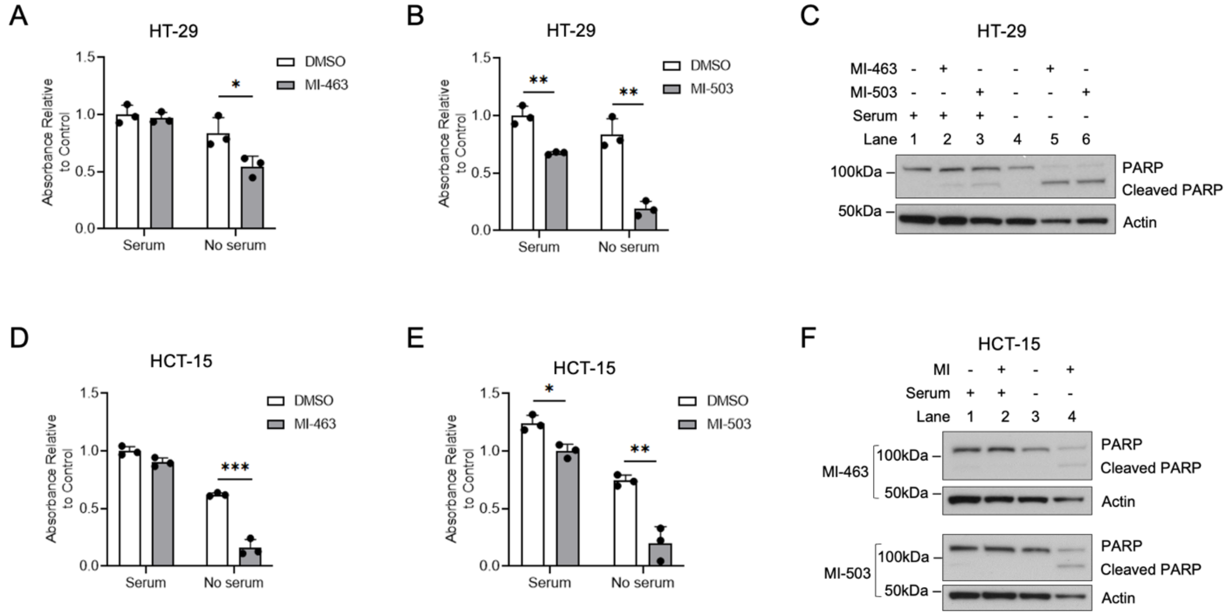

Supplementary Figure S4. Menin inhibition synergistically suppresses CRC cells under serum starvation. A-C) HT-29 cells treated with 2.5 µM MI-463 or 2.5 µM MI-503 for 96 hours in serum containing (10% FBS) or serum-free media with assessment of cell growth by MTS assay (A/B) and PARP cleavage after treatment for 72 hours (C). D-F) HCT-15 cells treated with 2.5 µM MI-463 or 2.5 µM MI-503 for 96 hours in serum containing (10% FBS) or serum-free media with assessment of cell growth by MTS assay (D/E) and PARP cleavage after treatment for 72 hours (F). * $p < 0.05$. ** $p < 0.01$. ^{ns} $p > 0.05$

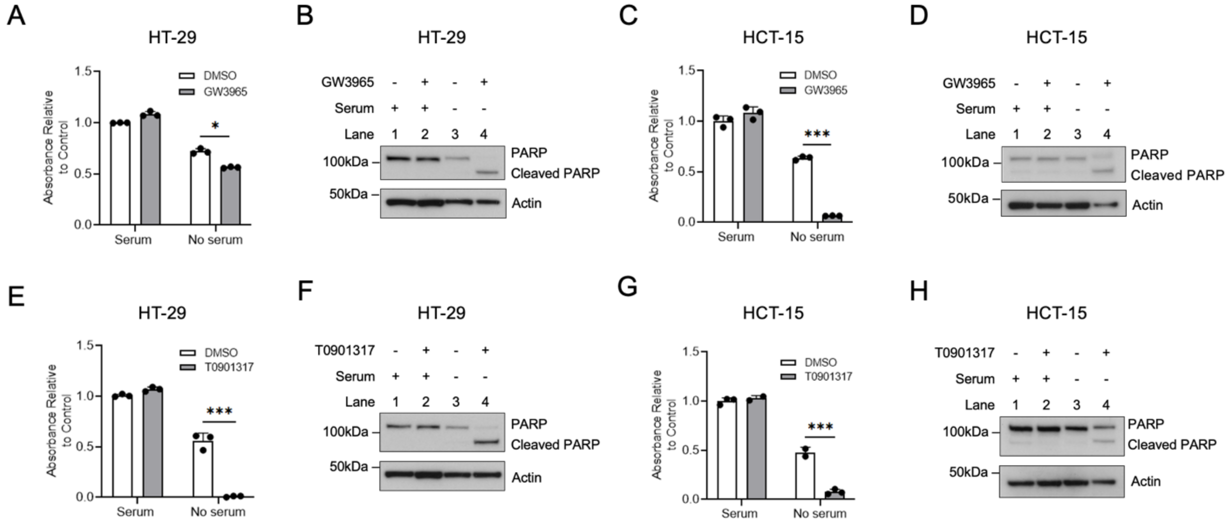

Supplementary Figure S5. Synergistic suppression of CRC cells by combined LXR agonist and serum starvation. A-D) HT-29 cells (A/B) or HCT-15 cells (C/D) were treated with 2.5 μM GW3965 for 72 hours in serum containing (10% FBS) or serum-free media, with assessment of cell growth by MTS assay and PARP cleavage. E-H) HT-29 cells (E/F) or HCT-15 cells (G/H) were treated with 10 μM T0901317 for 72 hours in serum containing (10% FBS) or serum-free media, with assessment of cell growth by MTS assay and PARP cleavage. * $p < 0.05$. *** $p < 0.001$.

Supplemental Table S1

Primers sequences utilized.

Gene	Experiment	Primer 5′ à 3′
Homo sapiens *MEN1*	RT-qPCR	F- GTGGCCGACCTGTCTATCAT<br>R- GTGCCTGTGATGAAGCTGAA
Homo sapiens *ACTIN*	RT-qPCR	F- GGTCATCACCATTGGCAATGA<br>R- GCACTGTGTTGGCGTACA
Homo sapiens *ABCA1*	RT-qPCR	F- TGTCCAGTCCAGTAATGGTTCTGT<br>R- CGAGATATGGTCCGGATTGC
Homo sapiens *ABCG1*	RT-qPCR	F- CCGACCGACGACACAGAGA<br>R- GCACGAGACACCCACAAACC
Mus musculus *Abcg1*	RT-qPCR	F- GAACCCGTTTCTTTGGCACC<br>R- GCAGATGTGTCAGGACCGAG
Mus musculus *Men1*	RT-qPCR	F- TGTGGGATGCCACCGC<br>R- ACGGTTGACAGCCAGGAAAT
Mus musculus *Gapdh*	RT-qPCR	F- TGCACCACCAACTGCTTAG<br>R- GGATGCAGGGATGATGTTC
Mus musculus *Men1*	Genotyping	F- CCCACATCCAGTCCCTCTTCAGCT<br>R- AAGGTACAGCAGAGGTCACAGAG<br>R- GACAGGATTGGGAATTCTCTTTT
Mus musculus *Vil1*-Cre	Genotyping	F- GCCTTCTCCTCTAGGCTCGT

		R- AGGCAAATTTTGGTGTACGG
LXRα shRNAs (1-3)	LXR Knockdown	1- GCAACTCAATGATGCCGAGTT<br>2- CCTTCCTCAAGGATTTCAGTT<br>3- GTGCAGGAGATAGTTGACTTT
ind-Menin shRNA1 Top Tet-On	Menin Knockdown	CCGGGCTGTACCTGAAAGGATCATACTCGAGTATGATCCTTTCAGGTACAGCTTTTT
ind-Menin shRNA1 Bottom Tet-On	Menin Knockdown	AATTAAAAAGCTGTACCTGAAAGGATCATACTCGAGTATGATCCTTTCAGGTACAGC
ind-Menin shRNA2 Top Tet-On	Menin Knockdown	CCGGGTGCAGATGAAGAAGCAGAAACTCGAGTTTCTGCTTCTTCATCTGCACTTTTT
ind-Menin shRNA2 Bottom Tet-On	Menin Knockdown	AATTAAAAAGTGCAGATGAAGAAGCAGAAACTCGAGTTTCTGCTTCTTCATCTGCAC
MEN1 sgRNA	Menin Inactivation	TGACCTGCACACCGACTCGC
